# MED12/376: Microbiology and the Hospital Intranet

**DOI:** 10.2196/jmir.1.suppl1.e55

**Published:** 1999-09-19

**Authors:** B Perera

**Affiliations:** ^1^Royal Oldham Hospital, OldhamUK

**Keywords:** Microbiology, Infection control, Intranet

## Abstract

**Introduction:**

With the roll out of the NHS computerisation programme, hospital intranets have come into operation in the last two to three years but wards and departments were unable to log on to the Intranet because of old / obsolete hardware that was still in use. Recently modern equipment has been installed at numerous sites, especially wards, making the intranet more accessible. Administrative and performance data were some of the first to make its appearance on the Intranet. Although laboratory results were available on computer, diagnostic and therapeutic information and guidance was not. In microbiology and infection control, there is a great need to get information across to the medical and nursing staff. Booklets containing information on how best to use the laboratory, and guidelines on the use of antibiotics are distributed to staff at the time of induction but these are soon lost.

**Methods, Results:**

Over two years ago it was decided to use the intranet at the Royal Oldham Hospital to provide information on microbiology and infection control. Folders, booklets, and other handouts distributed at induction of staff may not be read, because of the vast amount of such material that has been handed out, or lost after a short period of time. Once the information is on the intranet it is available throughout the hospital at any time of the day or night, and should be of value to doctors, nurses and other hospital staff. The first microbiology site was established in 1997 and contained a guide to Anti-microbial Therapy. Subsequently the following have been added:

1. Junior doctors Handbook (microbiology).
2. Microbiology Laboratory users Handbook.
3. Management of patients with MRSA.
4. Management of bite wounds.
5. Containers for specimens

It is planned to have all the necessary policies and protocols relating to microbiology and infection control on the Intranet.

**Discussion:**

Providing accurate information especially on infection control is a major activity of this department. Books, folders, leaflets, notices placed in strategic positions have all been tried with some success. Accessibility has been a problem whenever the information has been required. The hospital intranet is an ideal medium for storing and disseminating information. So far this has not been exploited by the medical / clinical staff. In addition to policies, protocols, and instructions for the management of patients the intranet can be used as a teaching tool in all areas of medicine. It is hoped that more hospitals will use this medium to improve the quality of the service provided. In hospitals where an intranet is available the additional cost of having your site would be minimal. In the long term it may be possible to reduce or even totally eliminate the hard copy folders, leaflets etc. that are costly to produce, distribute and maintain, and extremely difficult to update once circulated.

